# Genome-wide identification and integrated analysis of the *FAR1/FHY3* gene family and genes expression analysis under methyl jasmonate treatment in *Panax ginseng* C. A. Mey.

**DOI:** 10.1186/s12870-024-05239-6

**Published:** 2024-06-14

**Authors:** Yang Jiang, Zixia Zeng, Gaohui He, Mengna Liu, Chang Liu, Mingming Liu, Tingting Lv, Aimin Wang, Yi Wang, Mingzhu Zhao, Kangyu Wang, Meiping Zhang

**Affiliations:** 1 College of Life Science, Jilin Agricultural University, Changchun, Jilin, 130118 China; 2 Jilin Engineering Research Center Ginseng Genetic Resources Development and Utilization, Jilin Agricultural University, Changchun, Jilin, 130118 China

**Keywords:** *Panax ginseng* C. A. Mey., *FAR1/FHY3* gene family, Methyl jasmonate (MeJA) response, Cis-acting element analysis, Gene expression patterns

## Abstract

Ginseng (*Panax ginseng* C. A. Mey.) is an important and valuable medicinal plant species used in traditional Chinese medicine, and its metabolite ginsenoside is the primary active ingredient. The *FAR1/FHY3* gene family members play critical roles in plant growth and development as well as participate in a variety of physiological processes, including plant development and signaling of hormones. Studies have indicated that methyl jasmonate treatment of ginseng adventitious roots resulted in a significant increase in the content of protopanaxadiol ginsenosides. Therefore, it is highly significant to screen the *FAR1/FHY3* gene family members in ginseng and preliminarily investigate their expression patterns in response to methyl jasmonic acid signaling. In this study, we screened and identified the *FAR1/FHY3* family genes in the ginseng transcriptome databases. And then, we analyzed their gene structure and phylogeny, chromosomal localization and expression patterns, and promoter cis-acting elements, and made GO functional annotations on the members of this family. After that, we treated the ginseng adventitious roots with 200 mM methyl jasmonate and investigated the trend of the expression of four genes containing the largest number of methyl jasmonate cis-acting elements at different treatment times. All four genes were able to respond to methyl jasmonate, the most significant change was in the *PgFAR40* gene. This study provides data support for subsequent studies of this family member in ginseng and provides experimental reference for subsequent validation of the function of this family member under methyl jasmonic acid signaling.

**Supplementary Information:**

The online version contains supplementary material available at 10.1186/s12870-024-05239-6.

## Introduction

FAR-RED-IMPAIRED RESPONSE 1 (FAR1) and its homologous gene FAR-RED ELONGATED HYPOCOTYL 3 (FHY3) are a group of homologous proteins originating from transposases that are widely present in most angiosperms and were initially identified in *Arabidopsis thaliana* as an essential component of the photosensitive pigment A (phyA)-mediated far-red light signaling pathway [1]. In addition, the FAR1/FHY3 gene family plays multiple roles in plant physiology and development, such as light signaling response, stress response [2], chlorophyll biosynthesis [3], ABA signaling [4], regulation of the biological clock and flowering time [5], programmed cell death, and reactive oxygen species homeostasis [6], among others. The *FAR1/FHY3* family genes contain three functional structural domains, including a C2H2-type zinc finger domain, a putative core transposase domain and a SWIM zinc finger domain [7]. Among them, the C2H2 zinc finger structural domain is capable of binding to DNA, and the core transposase structural domain and SWIM structural domain are required for homo- or heterodimerization and transcriptional activation activities [2, 8–10]. So far, the *FAR1/FHY3* gene family has been identified in several plant species, *Arabidopsis thaliana* [11], *Populus trichocarpa* [12], *Camellia sinensis* [13] and *Solanum tuberosum* [14], but it has not been reported in ginseng.

Ginseng (*Panax ginseng* C.A. Mey.) is a precious perennial medicinal plant with a long history in use in the East Asian region. Ginsenoside is one of the secondary metabolites of ginseng and is its main pharmacological active ingredient, belonging to triterpenoids, which can be categorized into protopanaxadiol type saponin (PPD), protopanaxatriol group saponin (PPT) and oleanolic acid type ginsenoside [15]. Ginsenosides have a wide range of pharmacological effects such as anti-inflammatory [16], antidiabetic [17], antitumor [18], amelioration of ischemic brain injury disorders [19], and neuroprotection [20], more than 200 kinds of saponins have been isolated from ginseng [21]. In recent years, ginseng has been used in the fields of tonic drugs, beverages, and cosmetics [22], but ginseng mainly relies on field cultivation, and its growth cycle is relatively long and easily affected by the environment, such as soil condition, temperature, humidity, pests and diseases, and light, etc. [23, 24], therefore, using modern biotechnological means to improve the content of ginseng saponin is an important research significance.

Phytohormones play an essential role in plant growth and development, response to external stresses and secondary metabolic pathways [25], especially in regulating the formation of active ingredients in medicinal plants, and the application of phytohormones is also one of the effective and sustainable ways to increase the yield and improve the quality of medicinal plants [26, 27]. Therefore, phytohormones are also often used as inducers to induce the expression of relevant genes in the ginsenoside biosynthesis pathway in order to increase the yield of ginsenosides [28, 29]. Jasmonic acid (JA) and methyl jasmonate (MeJA), collectively called JAs, are important signaling molecules in plants [30] and play a vital role in plant growth and biosynthesis of secondary metabolites. Both the treatment of exogenous JAs and the changes of endogenous JAs produced by the plant itself in adversity can affect the formation and accumulation of triterpenoids [31, 32], and relevant studies have confirmed that exogenous application of MeJA can induce changes in triterpenoid saponin content and the expression of genes related to triterpenoid synthesis in the adventitious roots of ginseng [33, 34], and at the same time, methyl jasmonate (MeJA) has the characteristics of a high and stable inducing effect, so we chose MeJA as an inducer to treat adventitious roots of ginseng in the study.

In this study, we used bioinformatics to screen and identify the *FAR1/FHY3* gene family in the ginseng transcriptome database, analyzed its gene structure, phylogenetic relationship, GO functional annotation, chromosomal localization, and performed expression pattern analysis based on the expression data of genes in this family in ginseng, as well as analyzed the promoter cis-acting elements, in addition to light-responsive elements, the *PgFAR1* gene also contains abundant hormone-responsive elements. By under 200 mM MeJA to treat the ginseng adventitious roots, we examined the expression trends of the four genes containing the most MeJA-responsive elements at different treatment times, and initially discussed the expression patterns of these family members under methyl jasmonate treatment, with a view to providing theoretical references and bases for the subsequent functional studies and applications of *FAR1/FHY3* genes in ginseng.

## Materials and methods

### Identification of the *FAR1/FHY3* gene family in ginseng

In this study, based on the ginseng transcriptome databases, the Hidden Markov Model (HMM) (Pfam: PF03101, PF10551, PF04334) of *FAR1/FHY3* gene was downloaded from the PFAM (http://pfam.xfam.org/) protein database, and the local HMMER software (http://hmmer.org/Download.html) in the Jilin Ginseng Protein Database to retrieve protein sequences containing the FAR1/FHY3 structural domains, with an e value of 1 × 10^–6^. The results were submitted to iTAK version 18.12 (http://itak.feilab.net/cgi-bin/itak/index.cgi) to exclude spurious sequences. Finally, 145 transcripts from 59 genes were identified by using NCBI CD-Search (https://www.ncbi.nlm.nih.gov/Structure/cdd/wrpsb.cgi) and SMART online tools (https://smart.embl-heidelberg.de/smart/) to ensure the presence of the FAR1/FHY3 conserved structural domains in the transcripts of the candidate genes, and finally named.

### Phylogenetic evolutionary analysis of the *FAR1/FHY3* gene family in ginseng

Three protein sequences of FAR1 from each of the two species of Arabidopsis thaliana and rice were downloaded from PlantTFDB version 5.0 (https://planttfdb.gao-lab.org). The MEGA-X [35] software was used to perform multiple sequence comparison between the six downloaded FAR1 protein sequences and the sequences with complete ORFs in the FAR1/FHY3 family in ginseng, and the phylogenetic tree was constructed by the maximum likelihood method based on the results of the comparison, with the parameters adopting the default values of the system, and the evolutionary tree was beautified by using the iTOL (https://itol.embl.de) online website.

### Analysis of gene structure and conserved structural domains of *FAR1/FHY3* gene family in ginseng

To understand the structure of these transcripts, the PgFAR1 protein sequences were uploaded to the online tool MEME (https://meme-suite.org/meme/tools/meme) for predicting conserved motifs, setting the maximum number of motifs found to 10, and leaving other parameters at default values. The structural domains contained in the *PgFAR1* transcripts were analyzed by NCBI's CDD tool and visualized the results by using TBtools version 2.0 [36].

### Chromosome localization and collinearity analysis of the *FAR1/FHY3* gene family in ginseng

Based on the gene location information and chromosomal information of ginseng FAR1/FHY3, the location of the *PgFAR1* transcript on the ginseng chromosome was visualized using the MG2C (http://mg2c.iask.in/mg2c_v2.1/) online tool. Selected transcripts with ≥ 100% similarity in matching regions were analyzed for intraspecific covariance, and *PgFAR1* transcripts on chromosomes with duplicated gene pairs were mapped with R language.

### GO annotation and functional classification of the *FAR1/FHY3* gene family in ginseng

By using Blast2GO version 5.0 [37] to annotate and categorize *PgFAR1* transcripts, the transcripts were classified into functional categories (Biological Process, Molecular Function, and Cellular Component) based on the results of the annotations and counted the number of transcripts annotated to different functions. The results were visualized using the online tool jvenn [38] (https://jvenn.toulouse.inrae.fr/app/example.html). The functional subclasses to which the *PgFAR1* transcripts were annotated at level 2 were visualized by R language.

### Expression analysis of the *FAR1/FHY3* gene family in ginseng

The expression data of *FAR1/FHY3* family transcripts were extracted from the ginseng transcript expression database in four ginseng roots of different ages, 14 different tissue of four-year-old ginseng and 42 farmer’s cultivar roots, and removed the transcripts with zero expression. Visualized using R language to analyze the expression pattern of this family of transcripts in ginseng.

### Analysis of promoter cis-acting elements of the *FAR1/FHY3* gene family in ginseng

In order to further explore the possible biological functions of the PgFAR1 gene, the 2000 bp promoter region upstream of the start codon was extracted for cis-acting element analysis according to the location of the gene on the chromosome, and the promoter sequences were entered into the online website PlantCARE [39] (https://bioinformatics.psb.ugent.be/webtools/plantcare/html/) to obtain the relevant cis-acting elements, filtered out as containing the light-responsive signaling elements and hormone-responsive elements, and then mapped by using the software of TBtools version 1.0 [36].

### The *FAR1/FHY3* gene family response under MeJA treatment in ginseng

The ginseng adventitious roots were used and provided in the experiment by Jilin Engineering Research Center for Ginseng Genetic Resources Development and Utilization. The ginseng adventitious roots (1 g) were inoculated in 250 mL triangular flasks containing 150 mL of liquid MS medium and incubated in a shaker at 22 ℃, 110 rpm for 21 days. On the 22nd day, we added 200 mM MeJA into the culture flask and set the treatment time as 0 h, 6 h, 12 h, 24 h, 36 h, 48 h, 72 h, 96 h, 120 h, and collected 3 biological replicates, where 0 h was the blank control group. Put the collected samples in liquid nitrogen for quick freezing and store them in –80℃ refrigerator for subsequent experiments.

### Extraction of RNA from treated samples and fluorescence quantitative PCR

We took 1 g ginseng adventitious root material from different treatment times, extracted total RNA from ginseng adventitious roots using the TRIZOL method, determined its concentration, and reverse transcribed it into cDNA using the SPARKscript II RT Plus kit (Shandong Sparkjade Biotechnology Co., Ltd., Qingdao, China). The *GADPH* gene was selected as the internal reference gene, and the reaction was carried out using UltraSYBR One-Step RT-qPCR Kit (Low ROX) (CWBIO, Beijing, China) and 7500 Real Time PCR System to detect the changes in gene expression of four genes containing the most MeJA-responsive cis-acting elements in the treated samples, including *PgFAR21*, *PgFAR27-04*, *PgFAR34-02*, and *PgFAR40* genes in response to the treatment with methyl jasmonate. The reaction system was 10 μL, including 5 μL of UltraSYBR mixture (Low ROX), 1 μL of template, 0.2 μL each of upstream and downstream primers, and 3.6 μL of ddH_2_O. The qPCR conditions were set as follows: 95 °C for 10 min, 95 °C for 15 s, 60 °C for 60 s, and 40 cycles. In order to ensure the accuracy of the results for samples with different treatment times, we set up three biological replicates and three technical replicates, and the final results were calculated using the 2^−ΔΔCt^ analysis method.

## Results

### Identification of the *FAR1/FHY3* gene family transcripts

A total of 145 transcripts containing the FAR1/FHY3 structural domain were identified in the ginseng transcriptome database, and these 145 transcripts were categorized into 59 *PgFAR1* genes, which were named *PgFAR01*—*PgFAR59*. Table S1 contains specific information about these transcripts, including transcript, gene ID, transcript length (bp), DNA sequences (5'—3'), ORF (bp/aa), and conserve domain. These nuclear sequences ranged from 201 bp – 3630 bp in length, with the shortest gene fragment being *PgFAR02* and the longest being *PgFAR44-01*, and their amino acid length sizes ranged from 27 aa – 877 aa.

### Phylogenetic analysis of the *FAR1/FHY3* gene family

**Fig. 1:**
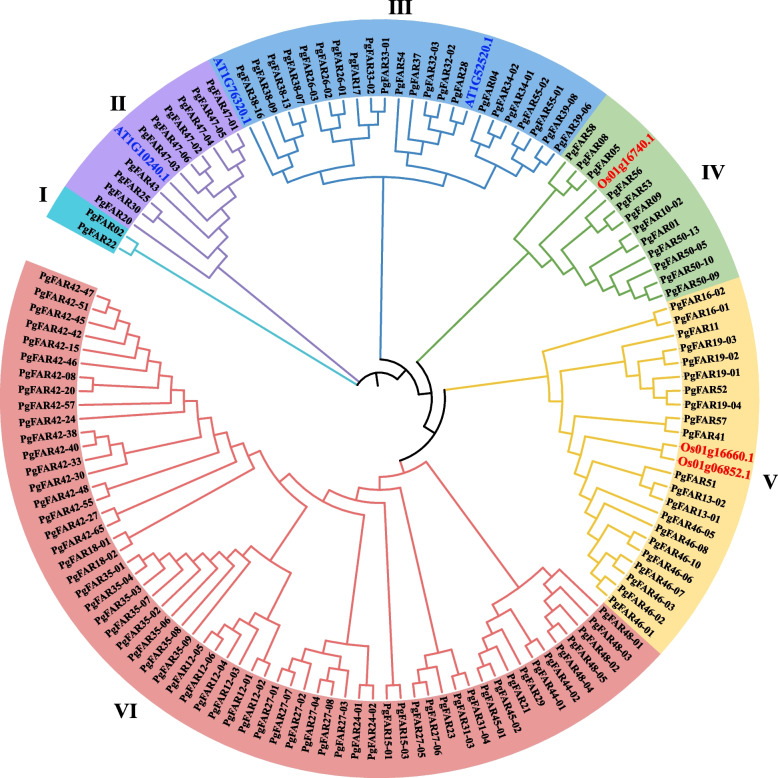
Phylogenetic tree of the *PgFAR1* gene family with exogenous species. The *PgFAR1* transcripts with an intact ORF in ginseng was used as the representative outgroup, and a total of six genes from *Arabidopsis thaliana* (*At*) and *Oryza sativa* (*Os*) as the outgroup. Proteins of the outgroup species are indicated by blue and red highlighted fonts. The subfamilies of the *PgFAR1* gene family are indicated using I, II, III, IV, V, and VI

We constructed a phylogenetic tree of ginseng FAR1/FHY3 protein sequences with the FAR1/FHY3 protein sequences of three from *Arabidopsis thaliana* (At) and three from *Oryza sativa* (Os). As shown in the Fig. 1, these members can be divided into 6 clades according to their relatedness. Clade I had the least number of members, including only 2 members, and clade VI has the greatest number of members, including 60 members. Among them, clade II and III were clustered with Arabidopsis FAR1/FHY3 protein sequences, and clade IV and V were clustered with rice FAR1/FHY3 protein sequences.

### Motif prediction and conserved structural domains of the *FAR1/FHY3* genes

**Fig. 2:**
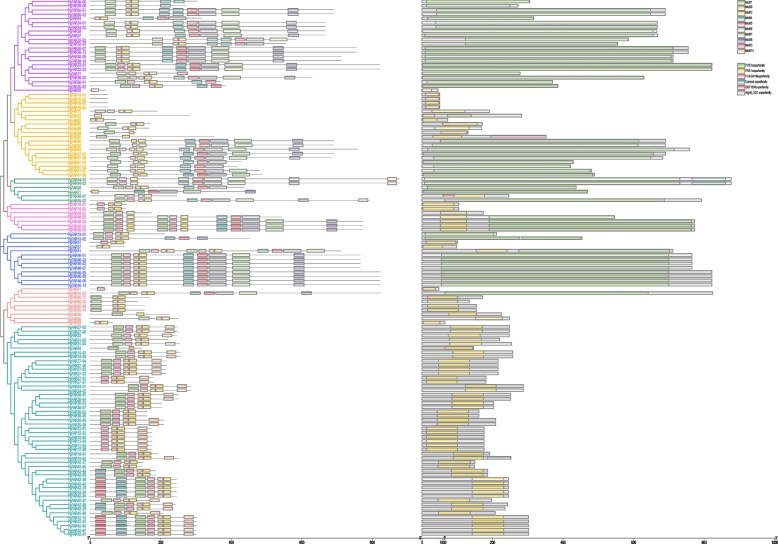
Phylogenetic tree, conserved motifs and conserved structural domains of PgFAR1. Different color fonts represent different subfamilies, and different color colored boxes represent different conserved motifs and conserved structural domains

An evolutionary tree of protein sequences of the FAR1/FHY3 family in ginseng was constructed, and Motif elements and conserved structural domains were also predicted and analyzed (Fig. 2). According to the evolutionary tree, the FAR1/FHY3 family in ginseng can be divided into seven subfamilies, and PgFAR45-02 in subfamily III contains 10 motifs. Compared with other subfamilies, the motifs of subfamilies V and VI are relatively simpler, suggesting that there may be functional differences among subfamilies of the ginseng *FAR1/FHY3* gene family. The results of the conserved domain analysis showed that except for PgFAR22, PgFAR41, PgFAR48-02, PgFAR48-03, PgFAR48-04, and PgFAR48-05, which contained both FAR1 and FHY3 conserved domains, the rest of the sequences contained only FAR1 or FHY3 domains.

### Chromosomal localization and gene duplication analysis of the *FAR1/FHY3* genes

**Fig. 3:**
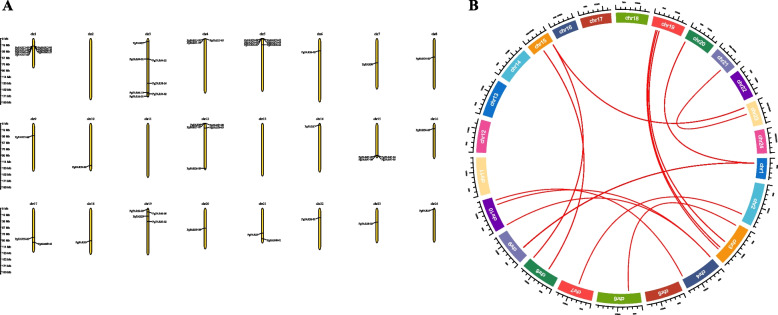
Chromosomal localization and collinearity analysis of the *FAR1/FHY3* gene family in *Panax ginseng*. **A** Chromosomal localization of the *FAR1* gene family in ginseng. **B** Collinearity analysis of *PgFAR1/FHY3* gene family members within the ginseng genome. The ends of the red arcs point to parallel pairs generated by gene duplication, the colored squares represent the chromosomes of ginseng, and the extrachromosomal scale indicates the length of the chromosomes

Based on the location information, 52 transcripts were localized to 24 pairs of chromosomes in ginseng. As shown in Fig. 3A the distribution of ginseng *FAR1/FHY3* genes on chromosomes was heterogeneous, with a maximum of 7 genes on chromosomes 1 and 3, and no *FAR1/FHY3 gene* family members were identified on chromosomes 2, 11 and 13. In addition, there was gene duplication in the distribution of the ginseng *FAR1/FHY3* gene family on the chromosome as shown in Fig. 3B.

### GO annotation and functional differentiation of the *FAR1/FHY3* gene family

**Fig. 4:**
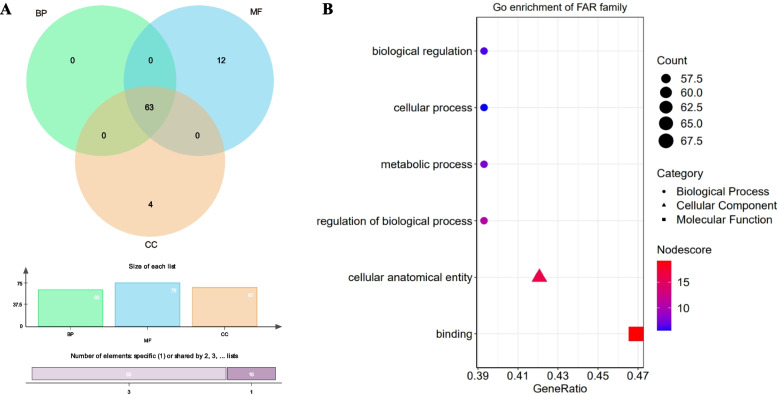
GO functional annotation and classification of *PgFAR1* transcripts. **A** Venn diagram of *PgFAR1* transcripts in Biological Process (BP), Molecular Function (MF), and Cell Component (CC) functional categories. **B*** PgFAR1* transcripts were annotated to 6 subcategories under GO functional level 2

To understand the function of each *PgFAR1/FHY3* transcript, we performed GO functional annotations on 145 transcripts, 81 transcripts were annotated to the Biological Process (BP), Molecular Function (MF) and Cellular Component (CC) categories in the GO database, and 64 transcripts were not available for annotation. Among the 81 *PgFAR1* transcripts annotated to function, 63 transcripts were noted to Biological Process, 75 transcripts were noted to Molecular Function, and 67 transcripts were noted to Cellular Component, with 63 transcripts annotated to three functions, 12 transcripts noted to Molecular Function only, and 4 transcripts noted to Cellular Component only (Fig. 4A). At level 2 (Fig. 4B), the gene was annotated to different nodes under three major categories: BP includes biological regulation (57), cellular process (57), metabolic process (57), regulation of biological process (57), MF includes binding (68), and CC includes cellular anatomical entity (61), thus suggesting that the *FAR1/FHY3* gene family is functionally differentiated and plays multiple functions in ginseng.

### Gene expression patterns analysis of the *FAR1/FHY3* genes in ginseng

**Fig. 5:**
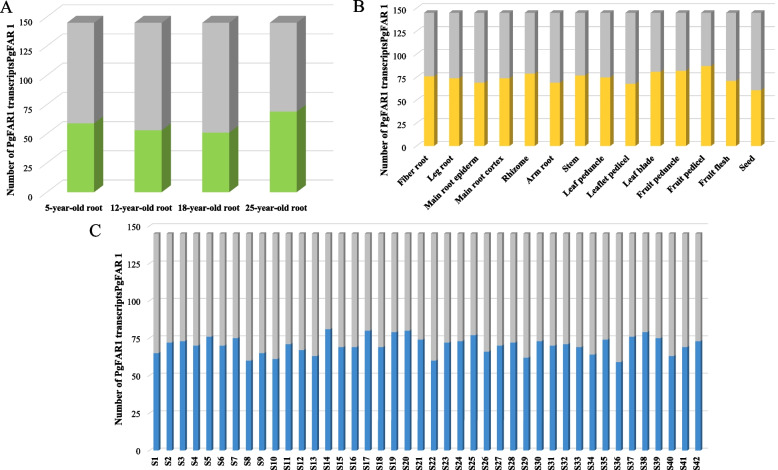
Expression numbers of *PgFAR1* transcripts in different ages, 14 different tissues and 42 farmers’ cultivars of ginseng. **A** The histogram of *PgFAR1/FHY3* transcripts expressed in 4 different year-old of ginseng roots. **B** The histogram of *PgFAR1/FHY3* transcripts expressed in 14 different tissues of 4-year-old ginseng. **C** The histogram of *PgFAR1/FHY3* transcripts expressed in 42 farm’s cultivars of 4-year-old ginseng

**Fig. 6:**
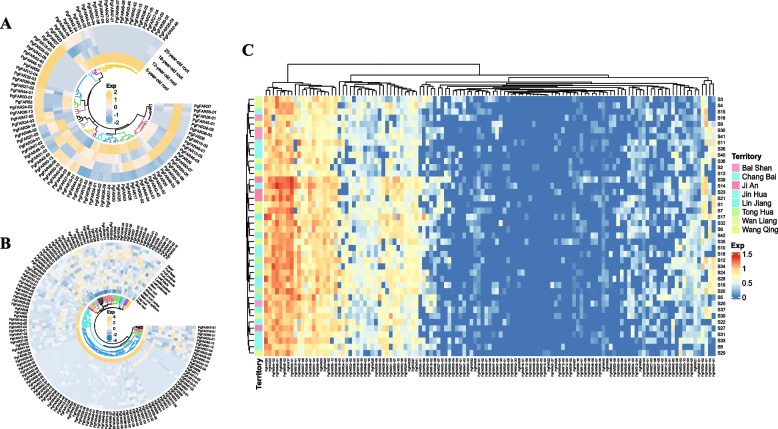
Heatmaps analysis spatiotemporal expression patterns of *PgFAR1* transcripts in *Panax ginseng*. **A** The *PgFAR1* gene transcripts expressed in the 4 different ages (5, 12, 18, 25 years-old) of ginseng roots. **B** The *PgFAR1* gene transcripts expressed in the 14 different tissues of 4-year-old ginseng. **C** The *PgFAR1* gene transcripts expressed in the 42 farmer’s cultivars of 4-year-old ginseng roots

In order to further understand the expression of *PgFAR1/FHY3* transcripts in ginseng roots of four different ages (5, 12, 18, and 25 years), 14 tissue of four-year-old ginseng (stem, fiber root, fruit peduncle, main root epiderm, fruit pedicel, rhizome, leaf peduncle, arm root, leaflet pedicel, leg root, leaf blade, fruit flesh, main root cortex, and seed), and in 42 farm cultivars (S1—S42), the expression data of *PgFAR1* transcripts were extracted for subsequent analysis (Fig. 5 and Fig. 6). From the expression of *PgFAR1* transcripts in ginseng roots of 4 different ages (Fig. 5A, Fig. 6A), 50 *PgFAR1/FHY3* transcripts were not expressed, 29 transcripts were expressed in ginsengs of four different ages, and 66 transcripts were expressed only in ginseng roots of specific ages. Among the 14 tissues of four-year-old ginseng (Fig. 5B, Fig. 6B), three transcripts were expressed in all 14 tissues, four transcripts were not expressed in any of the 14 tissues and the highest number of transcripts was expressed in Fruit pedicel of ginseng. Among the 42 farm cultivars (Fig. 5C, Fig. 6C), 25 transcripts showed expression and 22 transcripts showed no expression, which indicated that there was temporal and spatial variability in the expression of *PgFAR1* transcripts.

### Analysis of cis-acting elements in the promoter of the *FAR1/FHY3* gene family

**Fig. 7:**
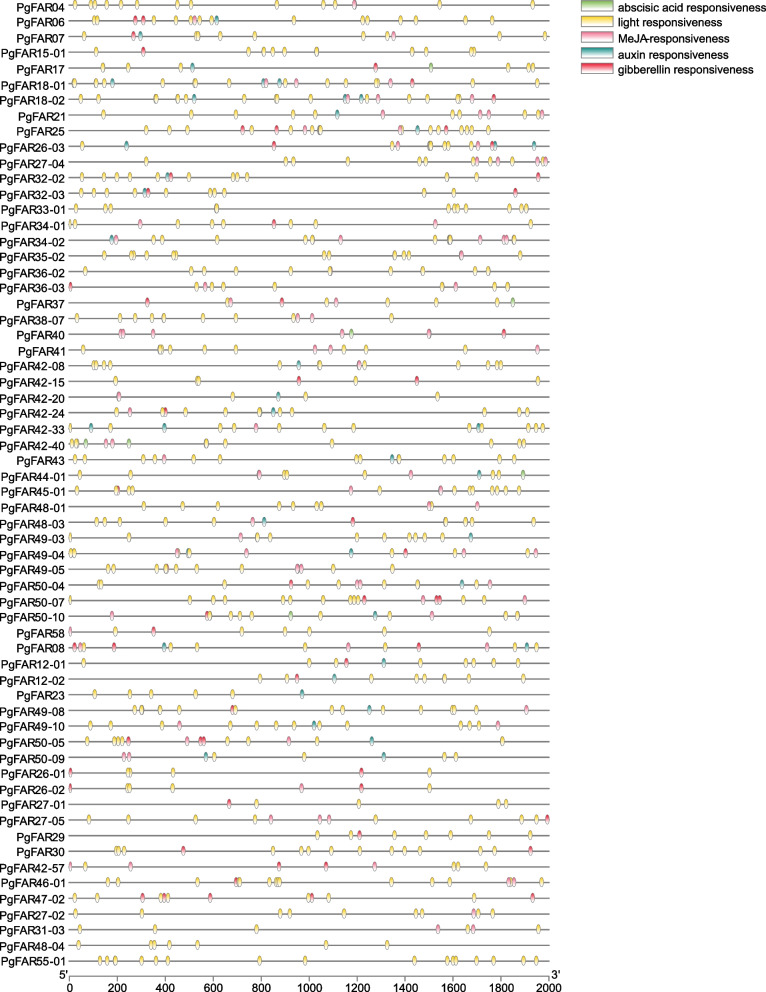
Analysis of cis-acting elements of the *FAR1/FHY3* gene family in ginseng. Circles of different colors show cis-acting elements with different functions

Usually the cis-acting element determines the function of the *FAR1/FHY3* gene in ginseng. The *FAR1/FHY3* genes in ginseng contains not only common cis-acting elements, such as TAAT-box and CAAT-box, and light-responsive signals, but also elements in response to low temperature, drought, defense, and stress, etc. In addition to these elements, they also contain a rich number of elements related to hormone response, which suggests that the *FAR1/FHY3* family may be involved in hormone signaling processes in ginseng and play an influential role in this process. Filtering for genes containing light-responsive elements and hormone-responsive elements yielded a total of 62 genes that met the conditions (Fig. 7). All 62 genes contained light-responsive elements. The hormone response elements were classified into 4 categories: auxin responsiveness; gibberellin responsiveness; abscisic acid responsiveness; MeJA-responsiveness. Thirty genes contained auxin responsiveness, totaling forty-one elements; thirty-nine genes contained gibberellin responsiveness, totaling sixty-one elements; forty-one genes contained abscisic acid responsiveness, totaling sixty-seven cis-acting elements; forty-five genes contained MeJA-responsiveness, totaling eighteen-eight elements. Among the hormone response elements, MeJA response elements were the most abundant. *PgFAR34-02* and *PgFAR40* genes contained ten MeJA cis-acting elements, *PgFAR21* and *PgFAR27-04* genes contained eight MeJA cis-acting elements, and these four genes contained the most MeJA response elements compared with the other genes, and therefore we selected these genes for the follow-up study.

### Expression analysis of *PgFAR1* genes under MeJA treatment

**Fig. 8:**
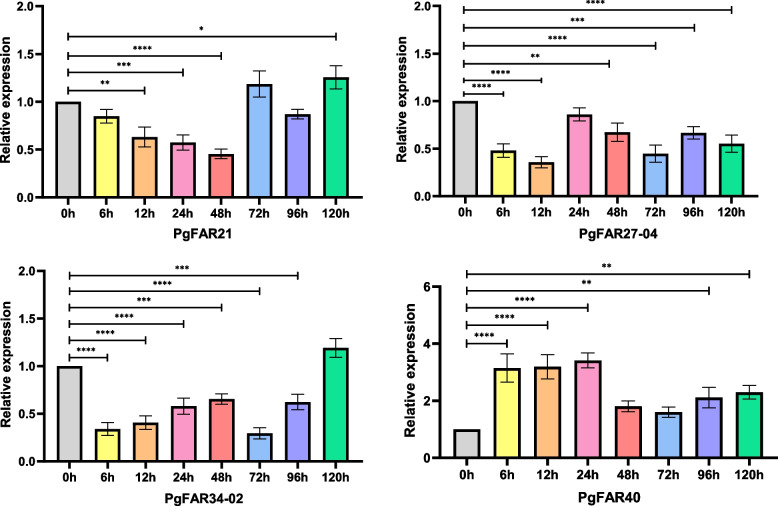
The expression of four *PgFAR* genes was detected under MeJA treatment of ginseng adventitious roots. The gene expressions of *PgFAR21*, *PgFAR27-04*, *PgFAR37-04* and *PgFAR40* genes were analyzed by qRT-PCR at different treatment times, and the gene expression at 0h was set as "1" to calculate the relative expression of the four genes. Values are the average of three replicates, “*” indicate significant difference at *p* ≤ 0.05, “**” indicate significant difference at *p* ≤ 0.01, “***” indicate significant difference at *p* ≤ 0.001, and “****” indicate significant difference at *p* ≤ 0.0001, respectively

After treatment with 200 mM MeJA, the relative expression of four genes in ginseng adventitious roots samples with different treatment times was detected, and all four genes were able to respond to MeJA treatment to different degrees. After MeJA treatment the qRT-PCR results showed in Fig. 8 the expression of *PgFAR21* gene decreased within 48 h, increased at 72 h, and reached the maximum at 120 h. Expression of the *PgFAR27-04* gene was decreased below that of the 0 h control. The expression of *PgFAR34-02* gene was lower than that of the untreated group at 0 h for 96 h, gradually increased from 6 to 48 h, and reached a maximum at 120 h, which was higher than that of the 0 h control group. Unlike the remaining three genes, the expression of *PgFAR40* was higher than that of the 0 h control group at different treatment times, reaching a peak at 24 h.

## Discussion

*Panax ginseng* is a prestigious traditional Chinese medicine with a long history of application, and ginsenosides have broad prospects for application and economic value [40]. In addition to its role in light signaling in plants, the *FAR1/FHY3* gene family has now also been clarified in *Arabidopsis thaliana* in plant growth and development [11], and the family is also engaged in the regulation of a variety of physiological processes in plants, including phytohormone signaling as well as biotic and abiotic stress responses [14].

We obtained 145 *FAR1/FHY3* transcripts under 59 genes by screening from the ginseng transcriptome database. Compared to other crops, model plants *Arabidopsis thaliana* (12) [41], *Camellia sinensis* (25) [13], *Solanum tuberosum* (20) [14], *Populus trichocarpa* (51) [12], and *Eucalyptus grandis* (33) [42]. Ginseng contained a higher number of *FAR1/FHY3* gene family members, which may be due to the fact that ginseng is a tetraploid plant and a gene duplication event occurred during the evolutionary process. Chromosomal localization analysis revealed that *FAR1/FHY3* family transcripts showed non-uniform distribution on the ginseng chromosome. Gene duplication events play a key role in the expansion of gene families [43], and the results of covariance analysis indicated that gene duplication events occurred in *FAR1/FHY3* family members during the evolution of ginseng. The GO functional annotation results showed that 57 (39.3%) of the 145 transcripts were annotated to the metabolic process of the biological process, suggesting that the *PgFAR1/FHY3* transcripts may play an effective role in the metabolic process of ginseng. Sixty-eight transcripts (46.9%) were commented to the binding function among the molecular functions, which implies that the *FAR1/FHY3* family in ginseng mainly exerts the function of binding DNA. 67 transcripts (46.2%) were annotated to cellular components. The above results demonstrate that the *FAR1/FHY3* gene family is functionally diverse in ginseng.

Analysis of the expression patterns of *PgFAR1/FHY3* transcripts in ginseng roots of four different age stages, 14 different tissues of four-year-old ginseng and 42 farm cultivars. The results showed that the expression of *FAR1/FHY3* transcripts was time-specific in four different aged ginseng roots, with some of the transcripts being expressed only in four aged ginseng roots and it was only with time that the expression of some of the transcripts appeared to accumulate. Among 14 different tissues, the largest number of *FAR1/FHY3* transcripts were expressed in Fruit pedicel, but gene expression was higher in Fiber root, the tissue-specific expression of which reveals that the family may play different functions in different tissue sites; From the three expression heat maps, it can be seen that the expression pattern of transcripts formed by splicing of the same gene may differ in ginseng roots of the same year, in ginseng roots of the same tissue, and in ginseng of different farm cultivars. Meanwhile, the expression patterns of genes under the same subfamily categorized by the phylogenetic tree may not be similar, indicating that the expression of most genes is relatively independent.

Promoter elements are associated with potential biological functions of genes, and through the analysis of cis-acting elements we found that *FAR1/FHY3* family members have a wide range of biological functions in ginseng. Particular *FAR1/FHY3* genes, in addition to responding to light signals, may be related to the defense and stress responses to drought, hypoxia, low temperature and circadian rhythms in ginseng, while the abundance of hormone-responsive elements suggests that this family may participate in hormone transduction processes in ginseng. Methyl jasmonate is an effective inducer of ginsenoside biosynthesis in cultured cells and ginseng adventitious roots [34]. In this study, methyl jasmonate was used to treat ginseng adventitious roots, and changes in gene expression of four genes containing the most methyl jasmonate response elements were measured in ginseng adventitious roots at different treatment times. The expression trends of the four genes were different, but all of them responded to the induction of methyl jasmonate to varying degrees, with the difference that the *PgFAR40* gene expression was the most significantly changed, with an increase in expression relative to the control in all cases. Meanwhile, the *PgFAR40* gene can be an important candidate for subsequent study of the molecular mechanism of action of this gene family under the corresponding MeJA regulatory mechanism.

## Conclusion

In this study, a total of 145 *FAR1/FHY3* transcripts under 59 genes were screened and identified from the ginseng transcriptome database, and using bioinformatics, we performed phylogenetic tree, gene structure and conserved structural domains, chromosomal localization and covariate analysis, GO functional annotation, expression pattern analysis and promoter cis-acting element analysis of this family. By using fluorescence quantitative PCR, the four genes of ginseng *FAR1/FHY3* family, which contain the most methyl jasmonate response elements, were detected and analyzed at different times of 200 mM methyl jasmonate treatment, and all four *PgFAR1* genes were able to respond to methyl jasmonate, the most significant change was in *PgFAR40*, which showed an upward trend. This study fills the gap on the study of *FAR1/FHY3* gene family in ginseng, lays the foundation for the subsequent functional study of this family, and provides certain data reference for the subsequent study of the expression and mechanism of this family of genes under the treatment of MeJA.

### Supplementary Information

Supplementary Material 1.

## Data Availability

All data generated or analyzed during this study are included in this published article. All plant materials are available through corresponding authors upon request.
